# Supplementary material for: Statistics of modal condensation in nonlinear multimode fibers
Source: Nat Commun. 2024 Feb 7;15:1149. doi: 10.1038/s41467-024-45185-3 (PMC10850069; doi:10.1038/s41467-024-45185-3)
Supplement: Supplementary file 1 — Supplementary Information [file 41467_2024_45185_MOESM1_ESM.pdf]

Statistics of modal condensation in nonlinear multimode fibers. Supplementary Information

Mario Zitelli[1*], Fabio Mangini[1] and Stefan Wabnitz[1]

[1*]Department of Information Engineering, Electronics and Telecommunications, Università degli Studi di Roma Sapienza, Via Eudossiana 18, Rome, 00184, RM, Italy.

*Corresponding author(s). E-mail(s): mario.zitelli@uniroma1.it; Contributing authors: fabio.mangini@uniroma1.it; stefan.wabnitz@uniroma1.it;

Supplementary Note A: Validation of the weighted BE law against independent experiments

In order to provide an independent validation of the weighted BE law introduced in Sec. 2, we referred to the tests performed in [1] whose experimental modal decomposition method appears particularly accurate, and whose data are published. Fig. 2b of that work is an experiment performed using 200 fs pulses at 1040 nm, propagating over 50 cm of graded-index (GRIN) fiber with 50 μm diameter; 14 modal groups were measured with differential eigenvalues ϵ_i ranging between 0 and 70671 m^{-1}; thermalization was obtained in the experiment with 52 kW peak power. The experimental mean modal power fractions $|f_i|^2$ are reported in Fig. 1; degenerate modes are supposed with same modal power fraction and eigenvalue.

Numerical fits were performed using the weighted BE, Eq. 2, and the RJ, Eq. 21 with $\gamma = 1$. The RJ fit provided $T = 400$ m^{-1} and $\mu' = -72600$ m^{-1}, equal to the values calculated in the original work; the RJ law appears following the distribution up to the 7-th modal group, but it does not follow correctly the distribution of the higher-order modes (HOMs); the fit accuracy

was $R^2 = 0.959$. The weighted BE fit obtained $T = 23300$ m^{-1}, $\mu' = -71100$ m^{-1} and $\gamma = 70.1$; the curve appears following the distribution up to the 14-th group order, with accuracy $R^2 = 0.998$.

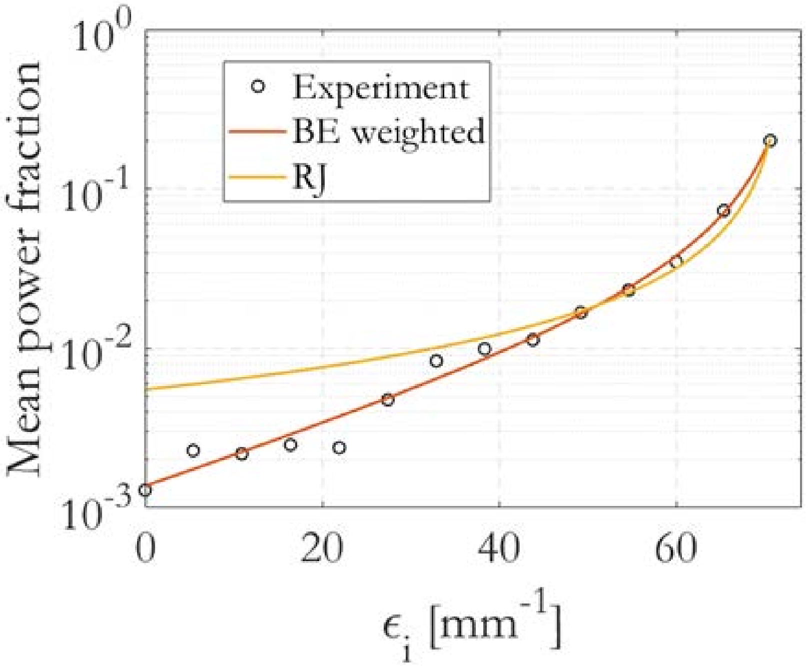

Fig. 1 Experimental mean modal power fractions from Fig. 2b of [1]. RJ and weighted BE fits are reported.

Figure 3e in [1] is a second experiment performed using 200 fs pulses at 1040 nm, peak power 20 kW, propagating in 2.5 m of GRIN fiber with 62.5 μm diameter. The measured modal groups were 28 with ϵ_i ranging between 0 and 176321 m^{-1}; the measured mean modal power fractions are reported in Fig. 2. The RJ fit provided in this case $T = 292$ m^{-1} and $\mu' = -178000$ m^{-1} with accuracy $R^2 = 0.938$; in the original work, values were 300 and -178000 m^{-1} respectively; the RJ is able to follow the experimental distribution up to the 8-th group order. The weighted BE fit obtained $T = 45700$ m^{-1}, $\mu' = -179000$ m^{-1} and $\gamma = 166$; the fit provided an accuracy of $R^2 = 0.976$ and it was able to overlap to the experimental distribution up to the 17-th group order.

A validation of the state equation Eq. 3 was performed calculating the error ϵ_{SE} from Eq. 4; the results were 0.64 % and 1.4 % for the two experiments, respectively.

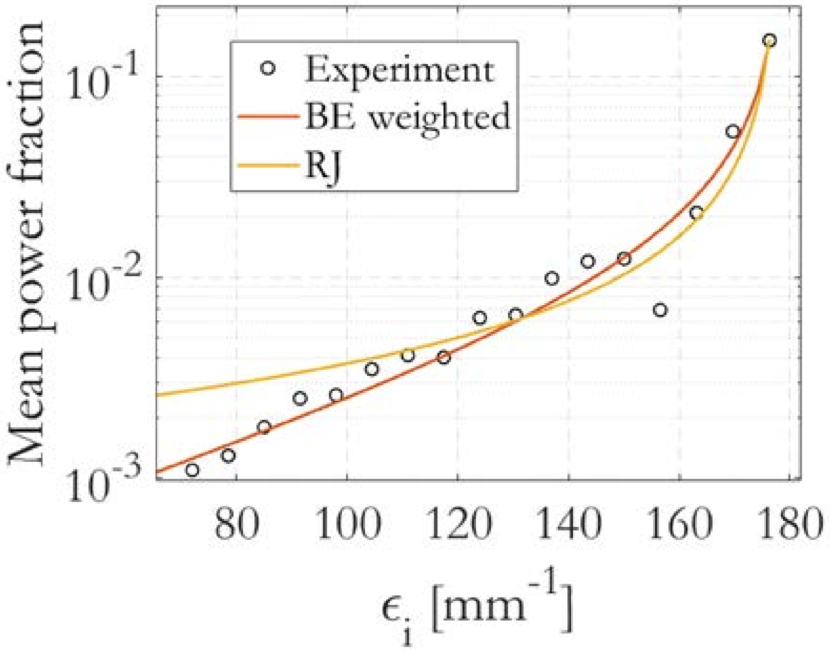

Fig. 2 Experimental mean modal power fractions from Fig. 3e of [1]. RJ and weighted BE fits are reported.

Supplementary Note B: Comparison of experimental modal distributions

In Fig. 3 it is reported a direct comparison of the modal distributions measured in Fig. 3 of the main text, at different pulse energies.

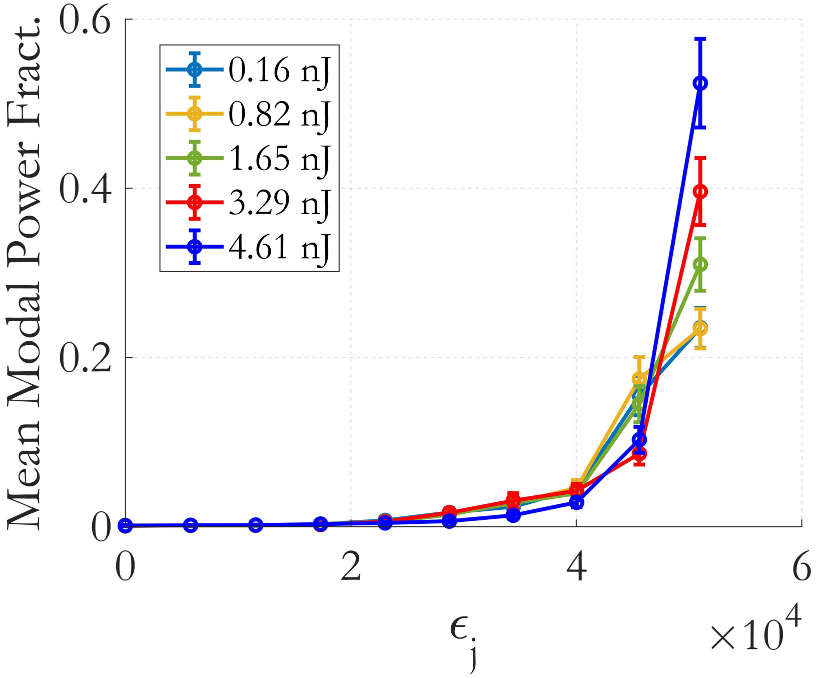

Fig. 3 Experimental mean modal power fractions from Fig. 3 of the main text, at different energy levels

Supplementary Note C: Comparison of simulated modal distributions

In Fig. 4 it is reported a direct comparison of the modal distributions simulated by following the method of section "Linear Disorder and Nonlinearity", with 28 propagated modes, over 100 m of fiber and at different pulse energies; weighted BE fits and the input distribution are also reported. At 5.0 nJ pulse energy, a train of solitons is obtained, with 95% of the power condensed to the ground state.

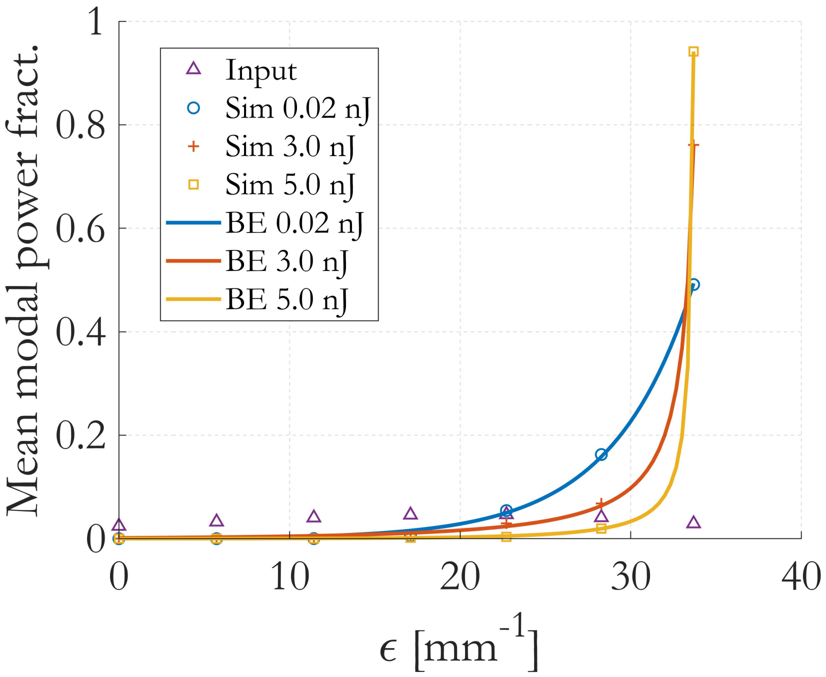

Fig. 4 Simulated mean modal power fractions, at different energy levels

Supplementary Note D: Experimental setup

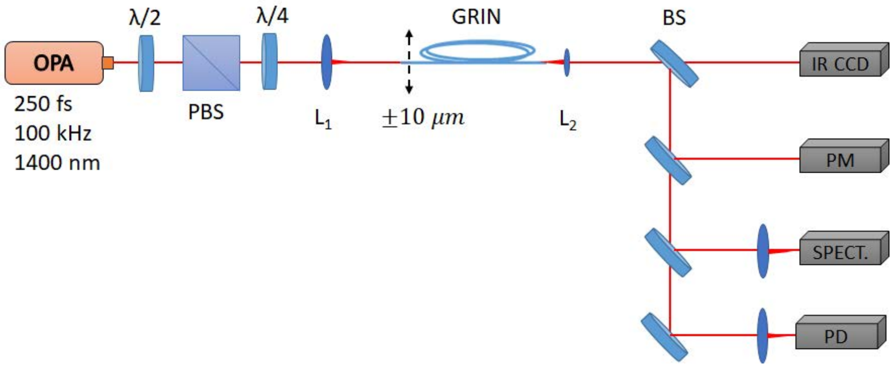

Fig. 5 Experimental setup. OPA: optical parametric amplifier; PBS: polarizer; $\lambda/2$, $\lambda/4$ half and quarter-wave plates; L_1: coupling lens; L_2: collimating microlens; BS: beam splitters; IR CCD: infrared camera; PM: power meter; SPECT: Spectrometer; PD: fast photodiode. GRIN fiber could be shifted by $\pm 10\mu$m at the input end.

Supplementary References

[1] Pourbeyram, H. *et al.* Direct observations of thermalization to a rayleigh–jeans distribution in multimode optical fibres. *Nature Physics* **18**, 685–690 (2022) .
